# Near‐Infrared‐Responsive Digital PCR‐Assisted Renal Cancer Exosomal miRNAs Insights and Regulation of Macrophage Polarization

**DOI:** 10.1002/advs.202509407

**Published:** 2025-08-29

**Authors:** Lexiang Zhang, Rokshana Parvin, Siyue Lin, Peiyu Chen, Gangling Wang, Dingmeng Hu, Ke Lin, Yun Cheng, Fangfu Ye, Bing Han, Gen Yang, Dexuan Wang

**Affiliations:** ^1^ Department of Pediatrics The Second Affiliated Hospital and Yuying Children's Hospital of Wenzhou Medical University Wenzhou Zhejiang 325027 China; ^2^ Key Laboratory of Structural Malformations in Children of Zhejiang Province The Second Affiliated Hospital and Yuying Children's Hospital of Wenzhou Medical University Wenzhou Zhejiang 325027 China; ^3^ Oujiang Laboratory (Zhejiang Lab for Regenerative Medicine, Vision and Brain Health) Wenzhou Institute University of Chinese Academy of Sciences Wenzhou Zhejiang 325000 China; ^4^ Joint Centre of Translational Medicine the First Affiliated Hospital of Wenzhou Medical University Wenzhou 325035 China; ^5^ Department of Biomedical Engineering Columbia University New York NY 10027 USA; ^6^ Beijing National Laboratory for Condensed Matter Physics Institute of Physics Chinese Academy of Sciences Beijing 100190 China; ^7^ Department of Orthodontics Cranial‐Facial Growth and Development Center Peking University School and Hospital of Stomatology Beijing 100081 China; ^8^ State Key Laboratory of Nuclear Physics and Technology School of Physics Peking University Beijing 100871 China; ^9^ Wenzhou Institute University of Chinese Academy of Sciences Wenzhou 325001 China

**Keywords:** digital PCR, exosome, macrophage polarization, microRNA, photothermal, renal cancer

## Abstract

Exosomes derived from tumors are critical agents in intercellular communication and the tumor microenvironment, offering a rich source of signatures for renal cell carcinoma (RCC) diagnosis. Conventional diagnostic techniques often suffer from limited sensitivity and can be invasive. This study presents an innovative approach using near‐infrared (NIR) digital PCR (dPCR) with black phosphorus‐embedded gelatin microcarriers for profiling exosomal miRNAs and modulating STAT3 signaling and macrophage polarization. Microcarriers produced via microfluidics, characterized by their phase‐change and photothermal properties, are subjected to thermal cycling using a custom NIR source. The study identified a 4.2‐fold increase in miR‐210 levels in RCC cells (ACHN and A498) compared to normal cells (HK‐2), with miR‐126 and miR‐30c levels decreasing by 7–9 times. Additionally, the method achieved a 20‐fold enrichment of miRNA‐34 in exosomes, leading to reduced STAT3 expression and decreased M2 macrophage polarization after co‐incubation. This pioneering dPCR method provides a robust tool for early RCC detection through exosomal miRNA profiling and opens new avenues for therapeutic exosome engineering. The study underscores the potential of dPCR‐based exosome genotyping in identifying cancer biomarkers and developing novel treatment strategies.

## Graphical abstract

A near‐infrared‐responsive digital Polymerase Chain Reaction technique with triple‐color coding profiles renal cancer exosomal microRNA biomarkers and their impact on macrophage polarization. Engineered miR‐34‐loaded exosomes via electroporation saturate molecular sponges, downregulating STAT3 in recipient macrophages to prolong M2 polarization. Stable microcarriers and optimized affinity capture boost renal cancer miR detection, with near‐infrared thermal cycling enabling programmable gene network intervention.

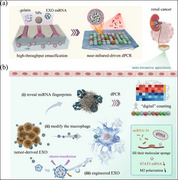

## 1 Introduction

Renal cell carcinoma (RCC) represents ≈3% of all cancers worldwide and remains associated with a high mortality rate of ≈40%, primarily due to late diagnosis and poor prognosis.^[^ ^1^ ^]^ While early‐stage RCC can often be effectively managed through surgery, a subset of patients still experiences recurrence.^[^ ^2^ ^]^ Traditional diagnostic methods, such as pathological biopsy and ultrasound imaging, remain the clinical standard. However, these techniques are either invasive, uncomfortable, or limited in sensitivity, and they may even risk promoting tumor dissemination.^[^ ^3^ ^]^ In recent years, exosomes—small, membrane‐enclosed vesicles secreted by most mammalian cells—have emerged as promising biomarkers. These vesicles carry a variety of bioactive molecules, including microRNAs (miRNAs), long non‐coding RNAs (lncRNAs), proteins, and lipids, which facilitate intercellular communication. Remarkably, a single tumor cell can release more than 10⁴ exosomes daily,^[^ ^4^ ^]^ making them abundant and potentially informative for disease monitoring. As a result, increasing attention has been directed toward the selective isolation and molecular characterization of exosomes. Advanced strategies such as aptamer‐guided fusion,^[^ ^5^ ^]^ thermophoretic aptasensors,^[^ ^6^ ^]^ and near‐field infrared spectroscopy^[^ ^7^ ^]^ have been employed to decode the heterogeneous RNA and protein content in tumor‐derived exosomes. Exosome‐based assays enable noninvasive classification of renal tumors, achieving 94.7% sensitivity in distinguishing clear‐cell RCC from benign lesions via CA9/CD147 immunocapture and profiling.^[^ ^8^ ^]^ Additionally, T cell‐inflamed gene expression profiles predict immunotherapy outcomes in clear‐cell RCC, with high‐expression cases showing a 42% improvement in progression‐free survival under pembrolizumab plus axitinib.^[^ ^9^ ^]^

Beyond their diagnostic value, exosomes have also shown potential as vehicles for targeted drug delivery^[^ ^10^ ^]^ They can influence both renal tissues and neighboring cell populations.^[^ ^11^ ^]^ Several engineering approaches have been explored to optimize exosome function, including the use of stem cell‐derived exosomes to promote kidney regeneration and modulate interactions between the ureteric bud and metanephric mesenchyme.^[^ ^10^, ^12^, ^13^ ^]^ In one strategy, electrical stimulation of transfected cells produced a large yield of exosomes loaded with therapeutic nucleic acids, resulting in a 50‐ to 1000‐fold enhancement of exosome release and mRNA payload, thereby amplifying antitumor effects.^[^ ^14^ ^]^ Despite these advances, many of the emerging techniques depend on complex instrumentation, hindering their widespread clinical application. Notably, exosomal RNA, protected by the lipid bilayer, can reflect early pathological changes, offering a window for disease intervention. For example, exosomal miR‐149 has been linked to renal injury, while miR‐193a, transferred from tumor‐associated macrophages, suppresses TIMP2 expression in RCC cells—highlighting new therapeutic angles.^[^ ^15^ ^]^ Unlike proteins, nucleic acids can be rapidly and sensitively amplified in vitro using widely accessible equipment. However, conventional methods for exosome isolation—such as ultracentrifugation, ultrafiltration, density gradients, flow cytometry, immunocapture, and microfluidic systems—are time‐consuming, equipment‐dependent, or lack specificity. A streamlined, bench‐top method is therefore urgently needed for RCC diagnosis and monitoring.

**Figure 1:**
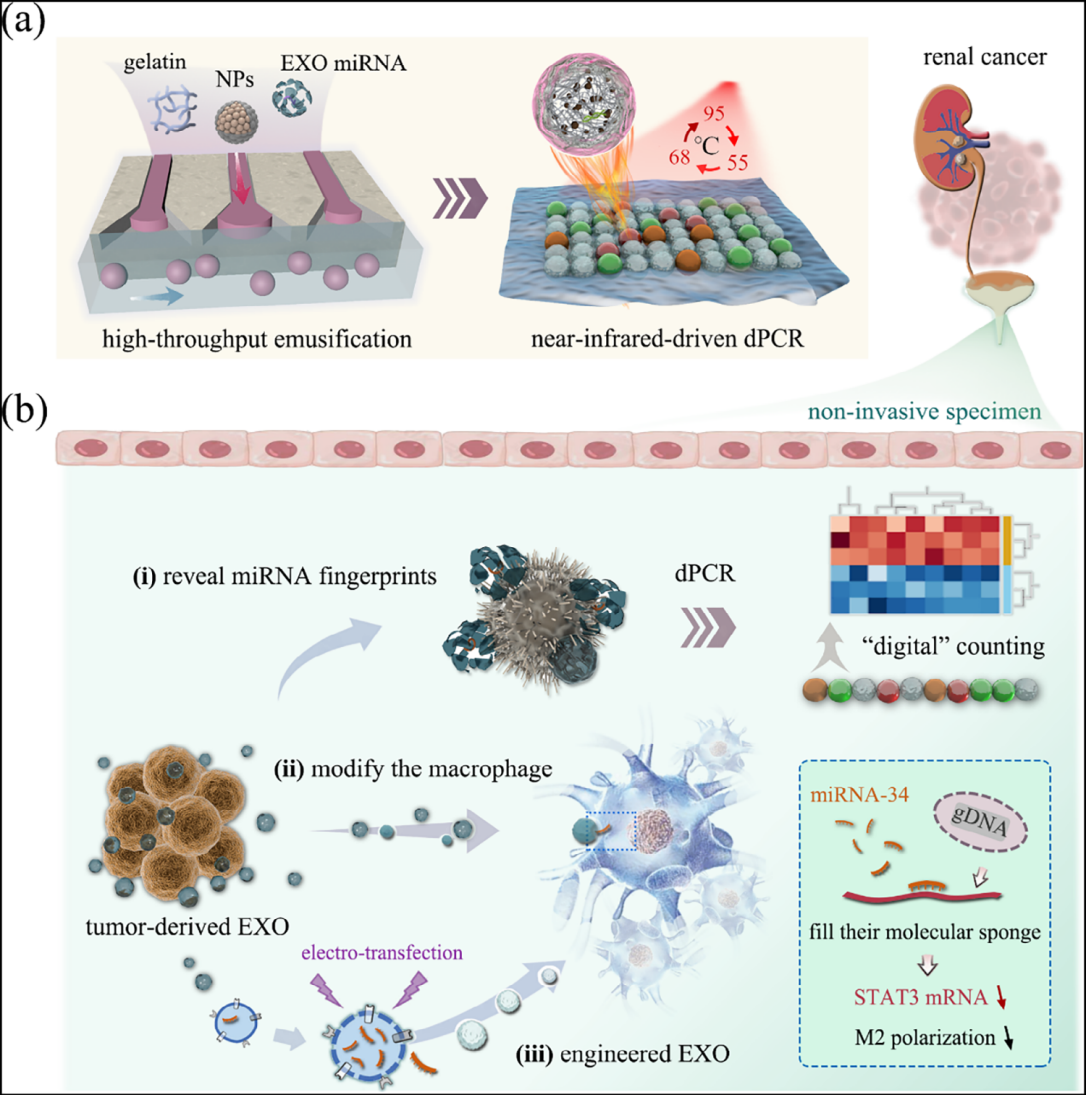
NIR‐responsive dPCR technique with triple‐color coding ability characterizes exosome endogenous microRNA biomarkers expression and its impact on macrophage polarization. Furthermore, the platform's capabilities are demonstrated through the optimization of engineered exosomes with a miRNA‐34 mimic, facilitating a reduction in Signal Transducer and Activator of Transcription 3 (STAT3) signaling by saturating their molecular sponge function, consequently prolonging M2‐type polarization of macrophages. For clarity, EXO is used as an abbreviation for exosomes.

To address these challenges, we propose a comprehensive workflow combining exosomal RNA profiling with engineering using black phosphorus (BP)‐based microcarriers activated by near‐infrared (NIR) light. These microcarriers enable digital PCR (dPCR) genotyping with high sensitivity and multiplexing capability, as illustrated in **Figure** **1**. dPCR, a recent advancement in nucleic acid amplification, enables absolute quantification by partitioning samples into thousands of individual reactions.^[^ ^16^, ^17^ ^]^ It offers superior sensitivity, inhibitor tolerance, and is particularly well‐suited for detecting early‐stage cancers.^[^ ^18^, ^19^ ^]^ The evolution of dPCR toward more intelligent and user‐friendly formats has accelerated its adoption in oncology,^[^ ^20^, ^21^, ^22^ ^]^ including applications such as microfluidic purification of tumor exosomes^[^ ^23^ ^]^ and immuno‐dPCR, which translates protein detection into barcoded nucleic acid readouts.^[^ ^24^ ^]^ Our team and others have developed thermally responsive substrates incorporating photothermal nanomaterials to enhance the precision of dPCR thermal cycling. Building on this, we integrate BP into temperature‐sensitive droplets to perform light‐controlled dPCR, offering a minimally invasive and highly sensitive method for early RCC detection.

In this study, we establish a platform that combines nonspecific exosome capture using nanoparticle (NP) probes with BP‐mediated NIR‐triggered dPCR. This approach quantifies exosomal miRNA levels and guides the engineering of exosome contents, particularly to modulate macrophage STAT3 signaling (Figure 1). Surface‐modified nanoparticles were designed to capture exosomes by utilizing an avidin‐biotin interaction,^[^ ^25^, ^26^ ^]^ which preserved the integrity of the exosomal RNA library. The BP‐functionalized microcarriers were fabricated by sequentially coating nanoparticles with BP, encapsulating them with silica, and emulsifying them with gelatin and PCR reagents in a microfluidic system with a millipede‐inspired design. These carriers served as thermally controlled reaction compartments. Upon NIR irradiation, the BP microcarriers efficiently converted light to heat, reaching 95 °C with adjustable response parameters. A customized NIR module enabled on‐site analysis, distinguishing RCC‐derived exosomal miRNAs from those of healthy controls. These miRNAs were linked to key oncogenic traits, including metastasis, drug resistance, and proliferation. Additionally, we explored how engineered exosomes could transfer specific miRNA cargos to macrophages, promoting a proinflammatory state. These findings underscore the potential of exosomes not only as diagnostic indicators but also as modulators of the tumor microenvironment.

## 2 Results and Discussion

### 2.1 Non‐Specific Exosomes Capturing Using Spiny Affinity Nanoparticles

**Figure 2:**
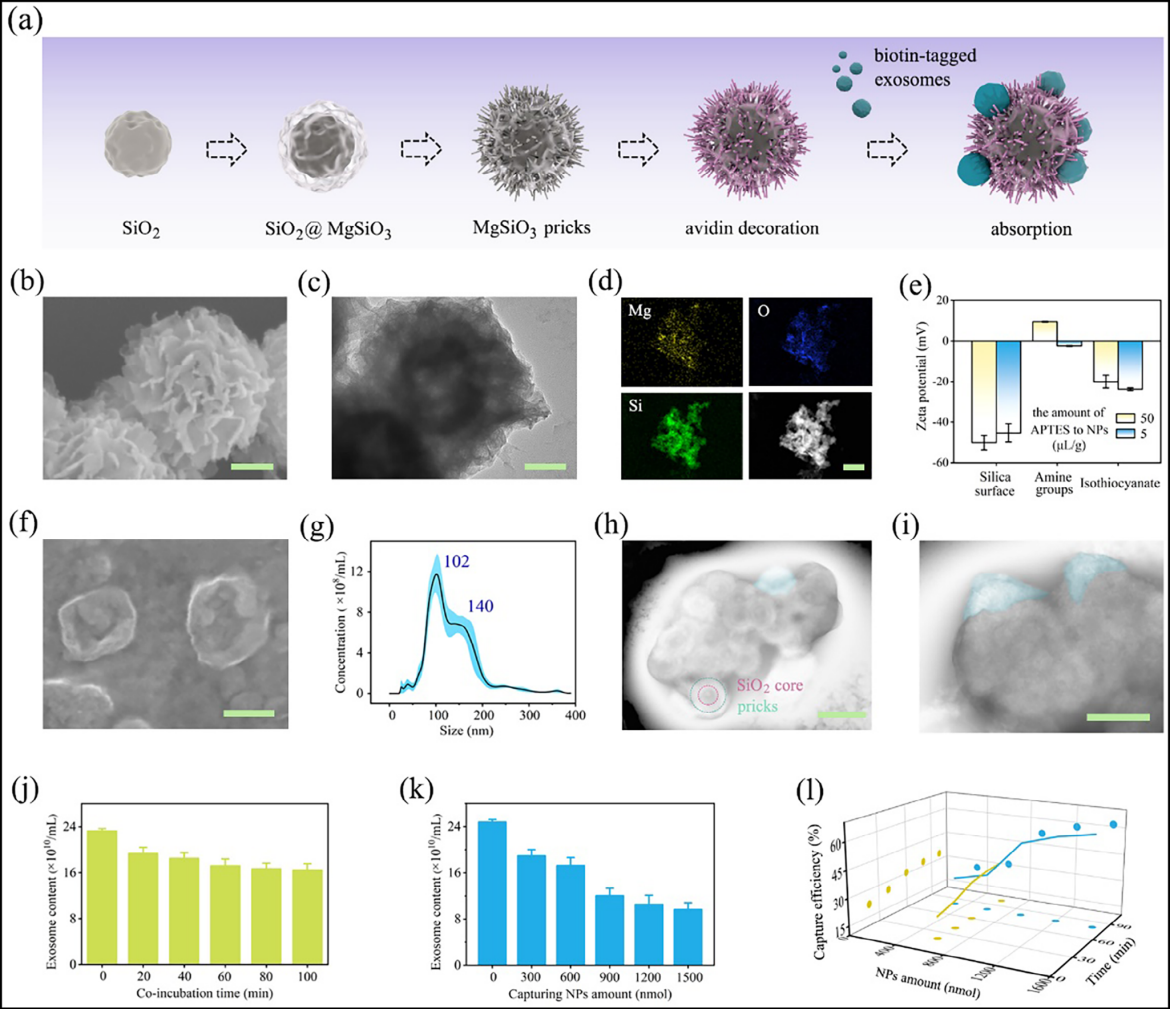
Investigation of NPs‐based nonspecific exosome capturing. a) Synthetic route of pollen‐like prickly NPs for exosome isolation. b–d) SEM and TEM images of SiO_2_ with generated MgSiO_3_ prick surface, with corresponding Mg, O, and Si elemental mapping analysis displaying composite assembly. e) Transformation of the zeta potential value according to the modification procedures. f) SEM image of saucer‐shaped exosomes in the model sample and g) their size distribution, where the blue band indicates the measured error bars. h,i) TEM images showing that exosomes were adhered onto the NPs. j–l) Exosome capturing efficiency assessed with varying NPs usage and co‐incubation time. Error bars, mean ± standard error of the mean (s.e.m.) (*n* = 3). Scale bars, 500 nm in (d,h, and i), 100 nm in (b,c), and 250 nm in (f).

To unravel a comprehensive RNA library within tumor‐derived extracellular vesicles, we attempted to nonspecifically isolate exosomes onto NPs utilizing a prickly surface and biotin‐avidin absorption. In a typical experiment, hierarchical synthesis and surface modification were performed as illustrated in **Figure** **2**a. Monodispersed SiO_2_ NPs with an average diameter of 336 nm (Figure S1, Supporting Information) were first prepared by modified Stöber approach, and then were incubated in ammonia solution at high temperature for separating out the silicate ions to combine with metal ions, preferentially generating silicates on the surface. As the reaction progressed, the silicate shell formed and grew gradually, resulting in surface‐prickly microspheres with a pollen‐like structure (Figure S1, Supporting Information).

Electron microscope and elemental analysis (Figure 2b–d) were employed to confirm their structures during the tunable fabrication process, indicating compact and firm arrangement of spines across the surface, yielding an average diameter of 400 nm. Magnesium elemental analysis revealed well‐overlaid pricks on the surface, with predominant presence of O and Si due to the SiO_2_ core and silica shell (Figure 2d). Notably, excess 1.2 mmol of MgCl_2_ resulted in abundant pinpricks, consuming excess silicate ions and hollowing out the NPs (0.05 g), while the addition of 0.2–0.5 mmol of MgCl_2_ led to an increasing trend of surface bumps with insufficient growth (Figure S1, Supporting Information). The pollen‐inspired NPs were subsequently avidin‐modified to capture biotin‐decorated exosomes. Based on the hydroxyl and other oxygen‐containing groups on the MgSiO_3_ shell, NPs were sequentially immobilized with amine groups, coupled with isothiocyanate, and finalized by avidin conjugation. Zeta potential shifting between positive and negative values were carefully measured to ensure adequate processing at each step (Figure 2e). Although the final particles carried a negative charge, insufficient amino modification impaired their ability to bind avidin.

To evaluate capture efficiency, exosome model samples containing ≈5 × 10^10^ population were isolated from cell culture media via ultracentrifugation, serving as reference standards for benchmarking the nanoparticle‐based extraction methodology. These vesicles exhibited a saucer‐shaped morphology, typically ranging from 30 to 200 nm in diameter (Figure 2f,g). DSPE‐PEG‐biotin‐labeled exosomes were blended with NP suspensions, resulting in a rapid and efficient capture of exosomes on the modified surface (Figure 2h,i). Subsequently, we explored capture efficacy as functions of key parameters, including applied NPs amount (ranging from 300 to 1500 nmol) and incubation duration (ranging from 20 to 100 min). The efficacy, derived from the exosome abundance in solution, correlated positively with these parameters, reaching a bottleneck ≈35–60% (Figure 2j–l). Further NPs addition and time extension yielded limited enhancement. Noteworthy is the improved capture efficiency afforded by the spiny biomimetic surface compared to our prior work utilizing bare NPs, enabling a more comprehensive transcriptome profile in exosomes under these optimized conditions. Taken this optimized condition together, we routinely extracted the cell‐derived exosomes from culture media, followed by electroporation treatment or extraction and analysis of nucleic acid cargos.

### 2.2 Precise Photothermal‐Mediated Temperature Control Substrate

**Figure 3:**
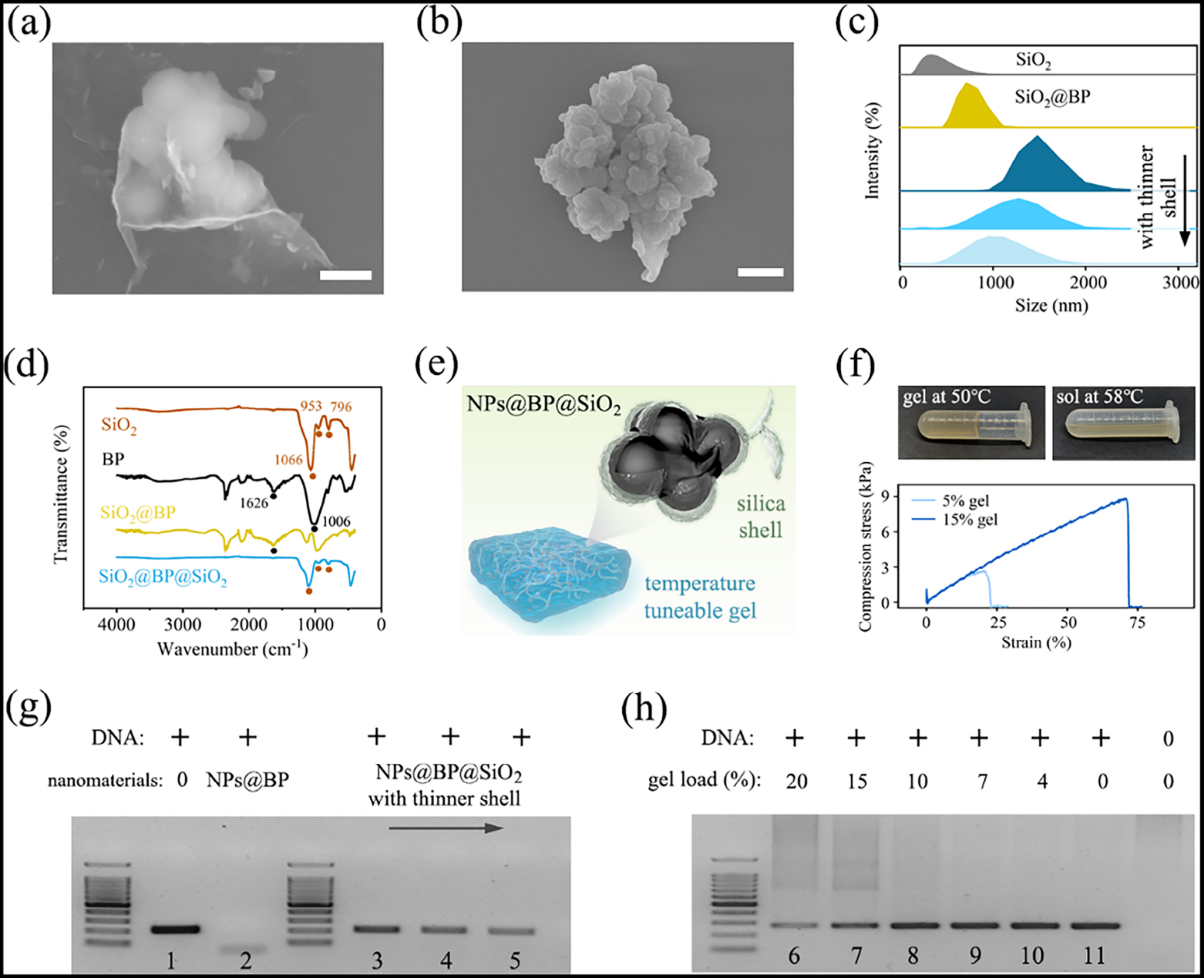
Characterizations of the photothermal nanocomposite‐doped gelatin as an NIR‐driven dPCR substrate. SEM image of a) SiO_2_@BP with nanofilm dispersed on the surface of dozens of SiO_2_ NPs and b) the final SiO_2_@BP@ SiO_2_. c) Size distribution along the synthesis progression. d) FTIR spectra of each compound and composite. f) Thermal‐reversible sol–gel transition of the nanocomposite‐doped gelatin and its compressive stress investigation. g) Electrophoresis gel images showing biocompatibility with different degrees of outer silica coatings. h) Electrophoresis gel images illustrating the amplification efficacy influenced by gelatin concentrations. Lanes 1–5 and 6–10 show results with the same amount of DNA input, respectively. Scale bar, 250 nm in (a) and 500 nm in (b).

For precise photothermal‐mediated temperature control, we engineered a NIR‐responsive nanocomposite. Leveraging the facile surface modification capabilities of SiO_2_ NPs, these were employed as distributing carriers to support BP nanosheets through polydopamine adhesion (**Figure** **3**a). The nanoscale dimensions of the NPs provided a high surface area for BP loading, enabling efficient NIR absorption while preventing BP accumulation and clogging at elevated temperatures (Figure S2a, Supporting Information). Subsequent silica coating via the Stöber method (Figure 3b) guaranteed essential biocompatibility with RNA, DNA, and enzymes. We optimized the silicon source amount and reaction time to form a complete and thin shell (Figure 3c; Figure S2b, Supporting Information), ensuring high light transmittance and maintaining the photothermal properties of the nanocomposite. Electron microscopy revealed the assembly of a dozen monodispersed BPs wrapped in nanofilms, reaching a size of 800 nm after assembling the shell.

Further characterizations through Fourier transform infrared (FTIR) spectrums unveiled hierarchical configurations and NIR‐absorbing ability (Figure 3d). Typically, the broad absorption band at 1006 cm^−1^ and small peak of 1626 cm^−1^ in BP were stemmed from P─O and P═O stretching vibration bands, respectively. The disappearance of these characteristic peaks implied complete silica coverage. Upon introducing the SiO_2_@BP@SiO_2_ nanomaterial, the resulting hybrid thermosensitive gelatin facilitated sol–gel transformation (≈52 °C) in synchrony with PCR activation upon NIR irradiation (Figure 3e,f). Gel electrophoresis with the presence of the nanocomposite validated that silica shells effectively prevented BP from affiliating nucleic acids and hindering PCR amplification (Figure 3g). A gelatin concentration of 7% (w/v) was selected as substrate according to PCR efficiency and mechanical property (Figure 3h).

### 2.3 Microcarriers Fabrication

**Figure 4:**
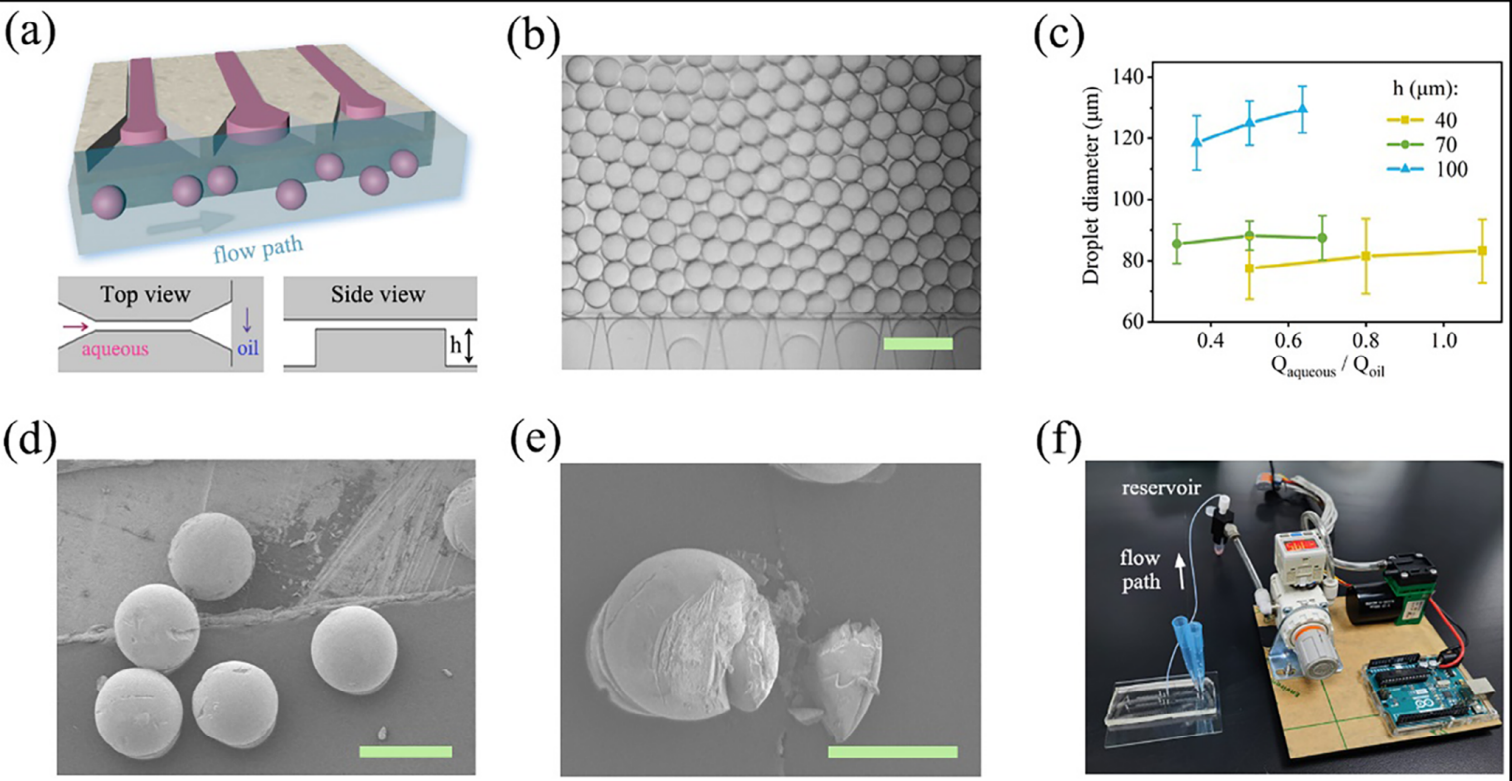
Microcarrier Fabrication and Performance for dPCR Usage. a) Two‐layer mask patterns and schematic views of constructed channel geometry. b) Photograph of the drop production process in parallelization operations. c) Investigation of droplet diameter as the function of the ratio of injected biphasic flow rates (Q_oil_ and Q_aqueous_ set in the ranges of 200–400 and 300–1100 µL h^−1^, respectively) and the steep height h. d,e) SEM images indicating the good sphericity and monodispersity of the gelatin microcarriers, with the sectioning view. f) Homemade vacuum‐pressure suction module connected to the chip outlet for droplet fabrication. Scale bar, 250 µm in (d) and 200 µm in (b and e).

For accurate nucleic acid detection via dPCR, we employed a millipede‐shaped step‐emulsification microfluidic device to split the reaction premix into high‐throughput microcarriers. The characteristic structure of poly(dimethylsiloxane) (PDMS)‐based chip was assembled through two consecutive photolithography processes (**Figure** **4**a), each with a step height of 40 and 70 µm, respectively. Tiny cross‐feature patterns (Figure S3, Supporting Information) drawn on both sides of the mask facilitated alignment during the exposures. Within each parallelized microchannel, the aqueous reaction fluid, containing gelatin, nanocomposite, and PCR solution, passed through a shallow triangular nozzle as an independent compartment for droplet formation. Subsequently, it fell off a stair into the oil fluid channel to complete the pinch‐off. This configuration, repeated 100 times in parallel, significantly accelerated the emulsification process to over 1 mL min^−1^ scale (Figure 4b). Evaluation of the biphasic flow rates, ranging from 200–1100 µL h^−1^, and the abrupt height h of the “step,” ranging from 50 to 100 µm, on droplet size uniformity revealed that droplet size was positively correlated with h but insensitive to operating flow rates (Figure 4c; Figure S4, Supporting Information), which was consistent with the previous results.^[^ ^27^ ^]^ Droplet diameter was tunable within the commonly used range of 70–130 µm, with a standard deviation less than 10%. A representative droplet array with a diameter of 116 µm ensured sufficient detection sensitivity and was adopted in this study, corresponding to ≈61 200 droplets derived from a 50 µL solution. The monodisperse morphology of droplets guaranteed quantitative measurement following Poisson statistics. To characterize this feature, gel microbeads were pre‐dried under supercritical ethanol conditions to prevent morphological distortion prior to electron microscopy, involving a stepwise ethanol dehydration process with concentrations increasing from 50 v/v % to 100%, each step lasting 30 min. Subsequent supercritical CO_2_ drying at 10 °C for 3 h preserved their structural integrity, with electron microscopy images (Figure 4d,e) confirming uniform sphericity and efficient nanomaterial incorporation in the representative morphology. To manage the small volume of reaction liquid (≈50 µL) and modularize microfluidic‐specific operations, a home‐made vacuum pressure regulation device was connected to the channel outlet to draw fluids in and form droplets with a ≈10^4^ Hz production frequency (Figure 4f).

### 2.4 Microcarriers Performance for Thermal‐Cycling

**Figure 5:**
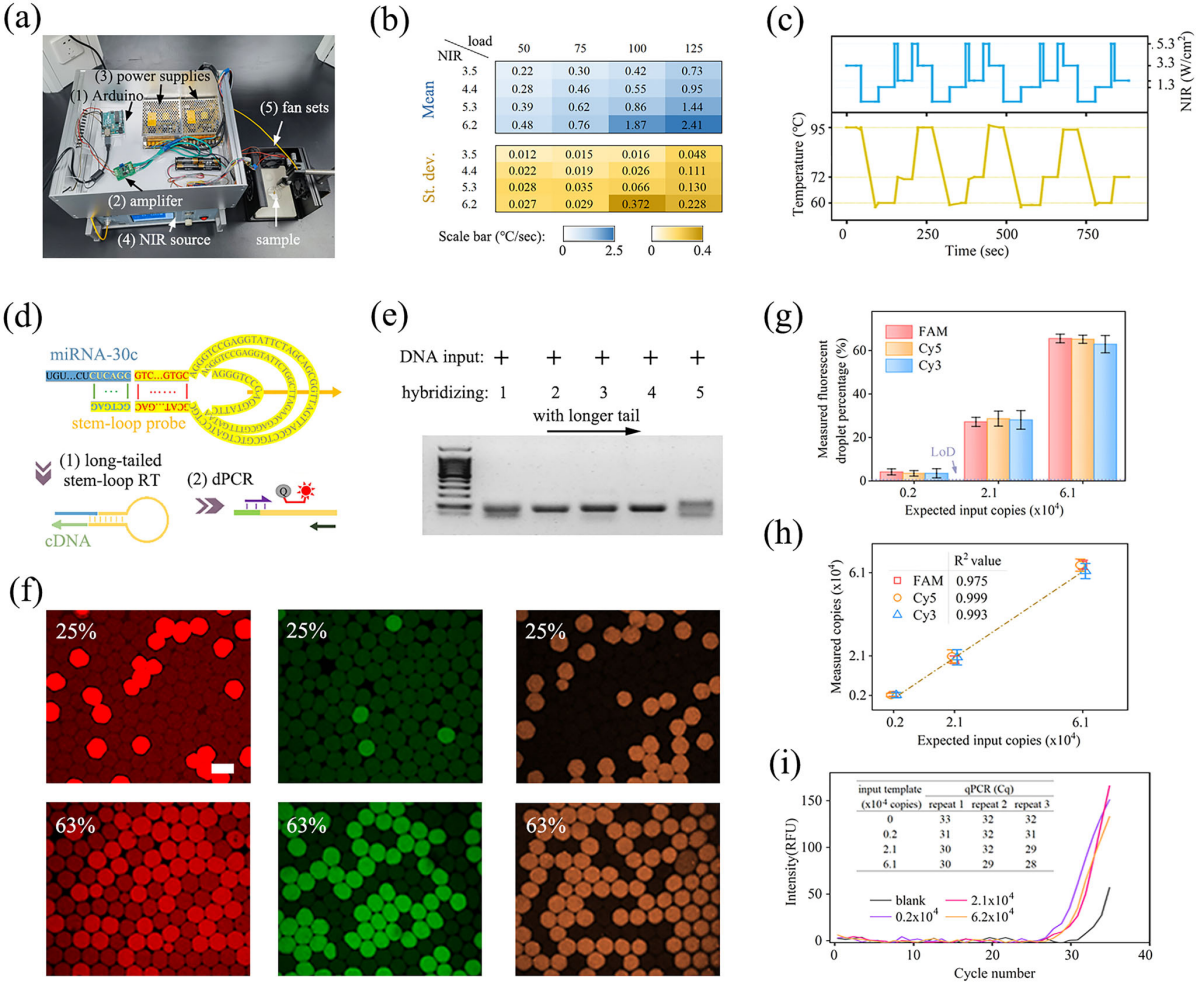
NIR‐responsive dPCR performance on detecting the miRNA of interest. a) Experimental layout of homemade programmable NIR radiation source and cooling fan sets for light‐driven dPCR automation. b) Temperature ramping up rate with different NIR intensities and nanocomposite concentrations using a 50 µL emulsion sample as a model. Mean and standard deviation (st dev) were derived from triplicate repeats. c) Four repetitive PCR cycles among 95, 60 and 72 °C embedded with tailored NIR irradiating steps in the Arduino program. d) Design of prolonged RT probes in the stem‐loop PCR scheme for primers and probes multiplexing. e) Electrophoresis gel band indicated successful amplification with 67–116 bp products. f) Representative fluorescence images showing post‐dPCR microcarriers at concentrations of 0–1 template per drop, using FAM, Cy5, and Cy3 TaqMan‐labeled testing assay, respectively. g) Experimental measured bright drop percentages varying with a range of expected input template copies in the triple color‐coded testing assays. h) Linearity of the deduced template abundance correlated to input amount. i) Quantitative comparison with qPCR approach based on the miRNA‐30 assay, where the Cq of 0.2 × 10^4^ copy condition was indistinguishable from the blank level. Scale bar, 100 µm in (f).

The system's thermal activation is driven by the superior photothermal conversion efficiency of BP nanosheets, as the heating performance of the SiO_2_@BP@SiO_2_ nanoparticles is primarily governed by the high BP loading within their core. The light‐to‐heat responsiveness of these microcarriers was investigated as a functions of NIR intensity and nanocomposite loading amount (**Figure** **5**a,b; Figure S5, Supporting Information). Remarkably, the temperature ramping‐up rate from 55 to 95 °C as the annealing step in PCR could be 1–2 °C/s with deviations often less than 8%. Knowing this, the assembly of a three‐temperature‐stage amplification was achieved through a sequence of NIR irradiations with corresponding intensities and durations. To convert the irradiation condition repeatedly and automatically, a homemade Arduino‐assisted NIR remote control module has been developed to mediate the customized PCR thermal cycles. The wiring diagram in Figure S6 (Supporting Information) illustrates the transmission of the virtual voltage command set by the Arduino program to the voltage amplifier, which was then output to the NIR laser source through the I/O port wiring. Microcarriers displayed sensitivity to NIR on/off and increasing/reduction conditions, maintaining a constant temperature responsiveness over four cycles (Figure 5c). These results highlight the repeatable and controllable performance of dPCR microcarriers in NIR responsiveness, laying the foundation for mediating temperature‐sensitive enzymatic bioreactions.

### 2.5 Profiling Dysregulated Exosomal miRNA Signatures

Evidence linking miRNA dysregulation to RCC progression prompted our investigation into a panel of crucial miRNA signatures derived from urinary cell exosomes using the photothermal dPCR technique. Among the selected miRNAs, miR‐126, a well‐established discriminator in numerous studies,^[^ ^28^ ^]^ exhibited significant downregulation alongside its target, vascular endothelial growth factor A. miR‐210, known for its role in HIF signaling, angiogenesis, and metabolism, displayed elevated expression in solid tumors, contributing to hypoxic conditions and poor outcomes.^[^ ^29^ ^]^ Consequently, miR‐210 emerged as a promising prognostic biomarker. miR‐30c, implicated in directing the HSPA5 or HSPD1 signaling pathways, demonstrated correlations with tumor progression. To accurately detect a set of miRNA molecules as short as 23 nt, we employed longer stem‐looped oligonucleotides compared to our previous work.^[^ ^30^ ^]^ This modification generated cDNA products of sufficient length (67, 81, 91, 101, and 116 bp as shown in Figure 5d,e) to facilitate the assignment of specific primers and probes for multiplexed and simultaneous detection. The resulting product bands confirmed the ability of a 14 bp neck ring structure to support an additional 30–65 nt loop without compromising hybridization efficiency. Consequently, we designed a list of specific stem‐loop probes, primers, and probes for each miRNA target (Table S1, Supporting Information).

Each miRNA target was individually labeled with FAM, Cy5, or Cy3 fluorophores so that fluorescing PCR(+) droplets indicated successful amplification of the target (Figure 5f). Conversely, nonfluorescent PCR(‐) droplets did not contain the target. To assess specificity and linearity, we progressively diluted the exosome elute, visually counting the percentage of bright drops. This approach allowed us to infer the template abundance in the droplet array according to Poisson loading statistics (Figure 5g,h; Table S2). For instance, enumerating 25% and 63% of the 61200 droplets fluorescing was essentially equivalent to randomly distributing 17 600 and 61 200 “targets” into 612 compartments, respectively. The bright drop percentage deviated from the linear relation to the serial dilution degree at high values due to a portion of PCR(+) droplets containing more than one template, as indicated by Poisson approximation. All three‐color assays exhibited good repeatability and fitting trends (R^2^ ≥ 0.975) over the average template‐per‐drop range from 0.05 to 1. The limit of detection (LoD) was determined as the level of fake bright drops in the blank sample. In a back‐to‐back comparison, our dPCR‐based method was capable of detecting 2000 copies and remained two orders of magnitude difference to the LoD (0.3% of bright drops), while the traditional qPCR method was challenging to distinguish (Figure 5i).

**Figure 6:**
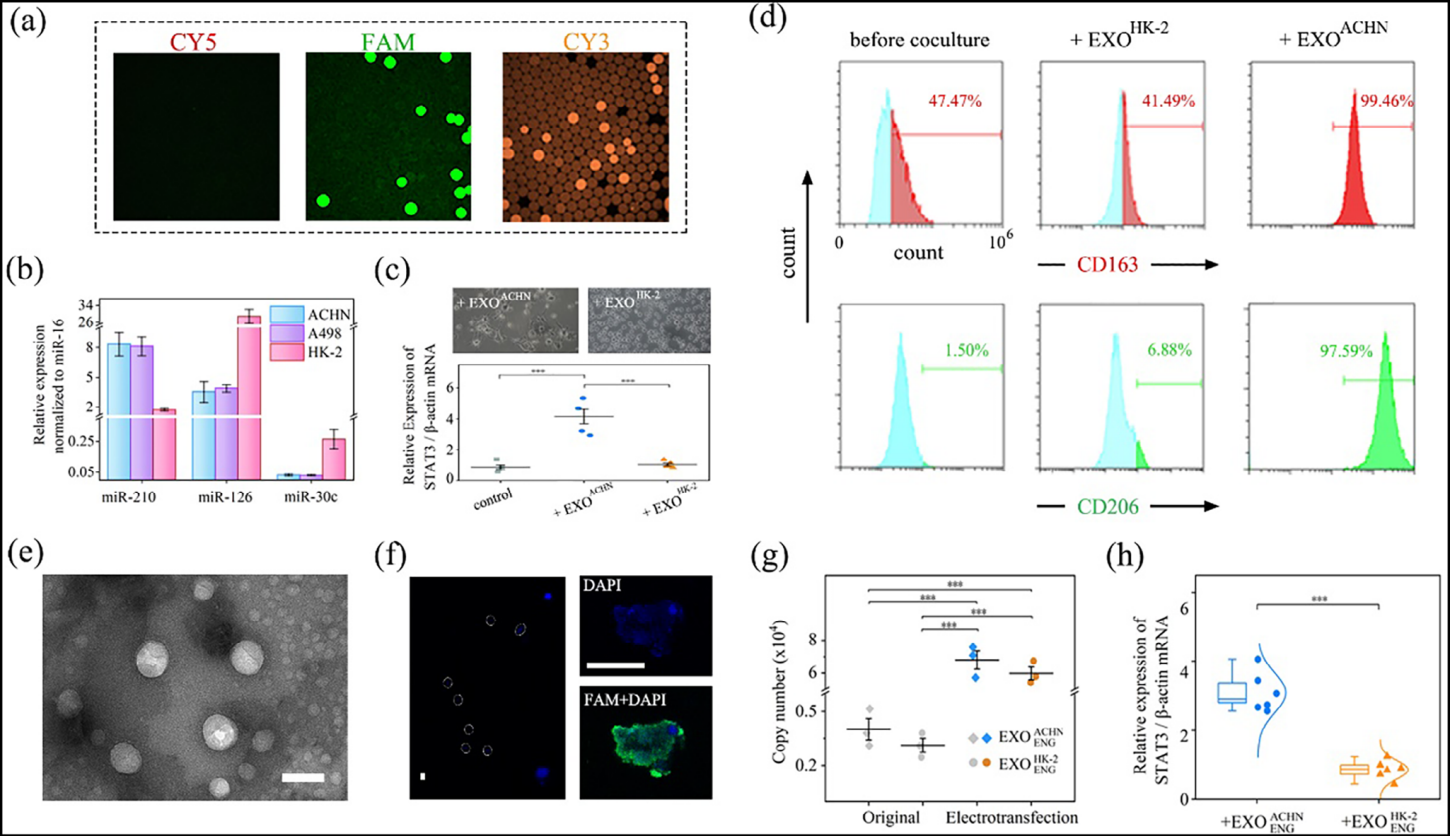
NIR‐responsive dPCR results for detecting a panel of renal exosomal miRNA and assisting in guiding exosomes to induce phenotypic changes of macrophages. a) Fluorescence images indicated simultaneous miRNAs detection via multiple color labeling. b) Relative expressions of miR‐210, miR‐126, and miR‐30c signatures among RCC and healthy cell sources. c) Representative images of macrophages states after coculturing with exosomes from human kidney cells ACHN and HK‐2, respectively. Evaluation of relative expression levels of STAT3 in macrophages before and after co‐culture with exosomes. d) Changes in CD163 and CD206 expression intensity of macrophages after co‐culturing with ACHN and HK‐2‐derived exosomes, respectively. e) TEM image indicated that exosomes remained intact morphology after electro‐transfection. f) Confocal microscopy image on left‐side showing uptake of cDNA fragments into exosomes by electro‐transfection, where the circled DAPI‐stained dots indicate localized enrichment of nucleic acids in the vesicles. Right‐side images show the DAPI staining intensity in the cytoplasm of macrophages also increased after co‐culture, possibly due to the stain‐labeled nucleic acids from endocytosed engineered exosomes. g) Changes in miR‐34 copy number in exosome samples with the same population after electro‐transfection. h) Engineered exosomes reduced the relative expression of STAT3 in macrophages. Scale bar, 100 nm in (e) and 10 µm in (f).

We classified them into a triplex dPCR panel to elucidate the relative expression levels of biomarkers in renal cancer cells (ACHN and 293T) and healthy kidney cells (HK‐2) secreted exosomes, transforming genotyping into a simultaneous characterization across three fluorescent channels (**Figure** **6**a; Figure S7, Supporting Information). Notably, miR‐126 and miR‐30c exhibited significantly lower expression levels in exosomes derived from tumor sources compared to those from healthy cells, while miR‐210 showed a substantial upregulation of 4.2‐fold (Figure 6b). The alteration in miR‐126 and miR‐30c showed decreases of 7.4–8.2‐fold and 9.1–8.7‐fold, respectively, in tumor sources. The small standard deviation and statistical significance not only validated distinct differences between cancer and healthy sources (Table S3, Supporting Information), but also underscored the dPCR's capability to identify minor changes. Our experimental findings aligned with reported trends,^[^ ^28^, ^31^, ^32^ ^]^ positioning these miRNAs as potential non‐invasive checkpoints for monitoring RCC progression.

### 2.6 Deciphering the Impact of Exosomes on STAT3 Signaling

In addition to accurately assessing miRNA fingerprints, extensive research has revealed that RCC‐derived exosomes activated the STAT3 pathway, influencing tumor growth and altering macrophage differentiation fate.^[^ ^33^ ^]^ However, quantitative studies on expression and regulation have been anticipated but not definitively illustrated. To address this, exosomes secreted by healthy renal cell HK‐2 and RCC cell ACHN were isolated and separately cocultured with M0‐type macrophages. Stimulated cells exhibited intrinsic small and roundish morphology, or evolved toward elongated cell bodies with stretched surfaces (Figure 6c). Flow cytometry test revealed significant expression of CD163 and CD206 only in the group treated with ACHN‐derived exosomes (Figure 6d), indicating their role in promoting macrophage M2 polarization. Based on this cell model, we conducted photothermal dPCR analysis to trace the mRNA expression level of STAT3 in macrophages, revealing a 4.8‐fold upregulation in the RCC‐exosome treated group, while it remained unchanged ≈0.9 in the control group (Figure 6c; Figure S8, Supporting Information).

Exosome‐macrophage interactions occur via complex systemic networks involving multiple signaling pathways. Among these, exosome‐transmitted lncRNA acted as a molecular sponge for miRNA‐34 to upregulate STAT3 expression and promote M2 polarization.^[^ ^34^ ^]^ Consequently, we hypothesized that infusing exosomes with miRNA‐34 mimics as a potential therapeutic vehicle could saturate its molecular sponge, tunably regulating STAT3 signaling and macrophage polarization. ACHN and HK‐2 cell‐derived exosome model samples, with a population ≈10^15^, were loaded with miRNA‐34 mimics via electroporation (Figure 6e). The transfection protocol was carefully optimized and involved blending an excess of miRNA and a 5 W voltage pulse for several seconds. Applying longer or greater voltage could easily generate foams, possibly due to vesicle damage. Given the difficulty in fluorescently labeling miRNA, we alternatively transfected 300 bp cDNA fragments into exosomes and visualized DAPI‐stained spots (Figure 6f), which might be attributed to the local enrichment of nucleic acids in exosomes. After degrading free‐floating miRNA mimic and deactivating the RNase enzyme, the results of photothermal dPCR measurement indicated 17–20 folds enhancement in loading efficiency, regardless of cell sources (Figure 6g).

Finally, M0‐type macrophages co‐cultured with these modified exosomes underwent our detection technique to monitor STAT3 mRNA signaling. The introduction of miRNA mimic into exosomes proved advantageous in suppressing STAT3 expressions, resulting in a 30% reduction in copy number compared to the baseline level of EXO^ACHN^ (Figure 6h,c). The discriminative ability of the test sets, featuring less than five‐fold changes, exhibited relative reliability. This robust discriminatory performance may arise from the saturation of miRNA‐34 over the lncRNA sponge from tumor exosomes, and enhancing its participation in the STAT3 pathway. Intriguingly, cells in this experimental group took an additional four days to manifest elongated morphological changes. In contrast, the engineering strategy brought no effect when co‐culturing with exosomes from healthy sources that have not induced macrophage polarization. This innovative design leverages NIR‐responsive dPCR as a suitable evaluation platform for guiding the delivery of exosome‐encapsulated biomolecule mimics, demonstrating promising potential in the development of nucleic acids therapeutics.

## 3 Conclusion

Over the past few decades, the field of disease diagnostics has rapidly progressed, driven by the integration of advanced sample preprocessing methods with cutting‐edge biomedical technologies.^[^ ^35^ ^]^ Innovations in responsive biomaterials and biomimetic systems have addressed long‐standing challenges and offered novel avenues for clinical application.^[^ ^36^ ^]^ As precision medicine becomes increasingly critical in RCC diagnosis, there is a growing need for platforms capable of accurately analyzing exosomal biomarkers. Such tools would not only enhance diagnostic accuracy but also provide insights for therapeutic monitoring and optimization. Within the tumor microenvironment, macrophages are key regulators of cancer progression. Their phenotypic flexibility allows them to either support anti‐tumor immunity or promote immunosuppression, often contributing to tumor growth.^[^ ^37^ ^]^ Of particular interest is the STAT3 signaling pathway, which plays a central role in macrophage polarization and is a potentialtarget in exosome‐based therapeutic strategies.^[^ ^38^ ^]^

To address these needs, we developed a non‐invasive, NIR‐responsive dPCR platform that leverages BP‐doped gelatin microcarriers and spiny nanoparticles with broad exosome affinity. These microcarriers combine the high sensitivity and resolution of microfluidic digital analysis with photothermally triggered thermal cycling. A vacuum‐driven droplet generation system and a programmable NIR light module were incorporated to enable automated, cost‐effective, and user‐friendly operation. Compared to traditional water‐in‐oil droplets, our gel‐based microcarriers offer superior stability, reduced evaporation, and minimized coalescence. Conventional PCR thermal cycling methods like Peltier elements, oil baths, and microwaves require substantial thermal capacitance for system components including reaction volumes, well plates, metal heating blocks, chips, etc. In contrast, the NIR heating strategy integrating 2D nanomaterials such as black phosphorus nanosheets in microcarriers enables precise temperature control with heating rates comparable to commercial PCR systems via NIR modules. Notably, our proposed scheme facilitates in‐solution heat generation, conferring advantages of low energy consumption, non‐contact thermal control, faster thermal response, and inherent compatibility with miniaturized integrated systems. This strategy synergistically combines the strengths of hydrogel‐based digital PCR and photonic PCR, leveraging the intrinsic detection performance afforded by dPCR—such as sensitivity and quantification accuracy.^[^ ^39^, ^40^ ^]^ In parallel, the use of microgel matrices permits spatial confinement and retention of amplified products at the single‐molecule level, facilitating downstream applications including flow cytometric sorting and high‐throughput library preparation. These integrated capabilities extend beyond the functional scope of current commercial dPCR platforms and reported photonic PCR systems.^[^ ^41^, ^42^ ^]^ These features position the platform as a robust tool for profiling oncogenic miRNAs in exosomes and studying complex regulatory pathways, including STAT3‐driven macrophage reprogramming in RCC.

In addition, our system was tailored to improve exosome loading with therapeutic miRNAs, addressing mechanisms such as lncRNA‐mediated sponging of miR‐34 in tumor‐secreted vesicles. Engineered exosomes were shown to suppress STAT3 signaling and modulate macrophage polarization, thereby disrupting the tumor‐supportive microenvironment. Overall, this platform demonstrates strong potential for uncovering molecular interactions and advancing exosome‐based diagnostics and therapies in renal cancer.

## 4 Experimental Section

### Materials

Single‐layer BP dispersion solution (2 mg mL^−1^) was purchased from Nanjing XFNANO Materials Tech. Gelatin (G7041), dopamine hydrochloride, mineral oil, Tween 20, Tris·buffer, and phorbol 12‐myristate 13‐acetate (PMA) were gained from Sigma. Avidin, DSPE‐PEG‐tagged Biotin, 3‐aminopropyltriethoxysilane, phenyldiisothiocyanate were gained from Life Technologies, Tanshui‐Tech., Beijing OKA, Diluent C buffer, Energy Chemical, and Shanghai Maokang Biotech., respectively. Anhydrous ethanol and hydrochloric acid were purchased Sinopharm Chemical. Tetraethoxysilane, pyridine, ammonia, magnesium chloride, and ammonia chloride were purchased from Xilong Scientific. PCR solution (RK20602), Bovine Serum Albumin (BSA), PCR and stem‐loop primers, and TaqMan probes were purchased from ABclonal, Biotopped, Shanghai Shenggong, and Shanghai Generay, respectively. FITC anti‐human CD206 Antibody and APC anti‐human CD163 Antibody were purchased from BioLegend. Polydimethylsiloxane (PDMS, 184), HFE 7500 oil, fluoroSurfactant, and connection tubing (PE2) were supplied from SYLGARD, 3 m Novec, RAN Biotechnologies, and Scientific Commodities, respectively. Ardunio (UNO) chip, voltage amplifier (0–10 V), power supply switch, and fan sets (5 V) were purchased from Seeedstudio, SLQXF, Wintech., and Studing, respectively. Deionized water utilized in this research was obtained from the Millipore Milli‐Q Plus system.

### Characterization

The morphology observation and elemental mapping were performed by TEM (FEI Tecnai G2 F20) and SEM (SU8010). Prior to SEM imaging, samples were sprayed with a platinum coating to enhance electroconductibility. Acceleration voltage was set ranging at 5–10 and 100–200 kV for SEM and TEM, respectively. Size distribution and Zeta potential value of materials were characterized and averaged through ten samplings by Nanophox Zetasizer (ZEN3600), with item abundance tuned ≈10^−3^‐1%wt. Exosomes size and population were deduced from hundreds of sequential snapshots obtained by Nanoparticle Tracking Analysis (Malvern NS300) with a syringe pump. Infrared spectra, UV–vis–NIR spectra, and Strain properties were measured by FTIR Spectrometer (Tensor II), Agilent (CARY5000), and Instron 5944 mechanical testing system, respectively. Microcarriers fabrication in the microfluidic chip was observed through an Olympus microscope (BX53) and fast camera (Optronis) setup. PCR, qPCR and gel electrophoresis were conducted by the Bio‐Rad equipment. Cellular surface biomarkers CD163 and CD206 were stained and measured by flow cytometer (Beckman Coulter). Fluorescent imaging of staining cells and post‐PCR microcarriers were characterized by Nikon A1 laser scanning confocal microscope with acquisition of bright field, FAM Cy5, and Cy3 channels.

### Cells and Exosomes Preparation

ACHN and A498 were cultured in MEM medium supplemented with 100 unit mL^−1^ penicillin, 100 µg mL^−1^ streptomycin, and 10% exosome‐depleted fetal bovine serum (HyClone) which was pretreated via 120 000 *g* ultracentrifugation at 4 °C for 12 h. HK‐2 were cultured under the similar conditions, except that the basal medium was replaced by DMEM. All cells were incubated at 37 °C and 5% CO_2_ in a humidified incubator. 45 mL of cultured medium was sequentially centrifuged at 300 *g* for 10 minutes, 2000 *g* for 10 min, and 100 000 *g* for 50 min to remove dead cells, cell debris, and macromolecules, respectively. After filtering, exosomes were harvested by the last step of 120 000 *g* centrifuge for 90 min. Meanwhile, total RNA was isolated from cells by commercial extraction kit (Vazyme). Macrophages were formed by stimulating THP‐1 cells (acute monocytic leukemia cell line, monocyte) with 300 ng mL^−1^ PMA in medium (RPMI‐1640) for 4 days, and replaced non‐PMA‐containing medium when cells were attached to the bottom. Cells were suspended in phosphate buffered saline (PBS) buffer, followed by incubation with anti‐human CD163 and CD206 antibody labeling dyes for 1 h in the dark, and subjected to flow cytometric analysis. Electrotransfection were conducted by Nucleofector 2b instrument. After successively degrading RNA (RNase A, R1030 Solarbio) and RNase enzyme (10701ES05, Yeasen), engineered exosomes underwent lysis or co‐culturing process.

### Synthesis of Spiny SiO_2_@MgSiO_3_ Nanoparticles for Exosome Purification

SiO_2_ NPs were fabricated by the modified Stöber approach. Next, 0.25 mmol of MgCl_2_·6H_2_O and 3.3 mmol of NH_4_Cl were introduced into a 10 mL aqueous solution, followed by the addition of 500 µL of ammonia solution (28%) and previously prepared SiO_2_ dispersion. The resulting mixture was maintained at 140 °C for 14 h, and then washed by ethanol. Then they underwent surface hydroxyl activation by immersing them in a 10 mL solution of 0.1% sodium hydroxide for 30 min, followed by thorough washing with water. To introduce amino groups onto the surface, a mixture containing 20 mg of SiO_2_@MgSiO_3_ NPs, 3 mL of concentrated ammonia, and 500 µL of aminopropyltriethoxysilane (APTES) was ultrasonically dispersed in 5 mL of absolute ethyl alcohol. The dispersion was stirred at 300 rpm for 3 h and subsequently washed sequentially with ethanol and deionized water. Then, a combination of 10 mg of NPs and 0.02 g of phenyldiisothiocyanate was added to 36 mL of dimethylformamide (DMF) containing 4 mL of pyridine. The NPs' surface was grafted with isothiocyanate after 2 h stirring. Throughout each step of surface modification, the Zeta potential of the NPs was regularly monitored. The alternation between positive and negative values was crucial, serving as an indicator of the success of the respective treatments.

### Nonspecific Isolation of Exosomes Using Functionalized NPs

A solution containing 0.002 g of DSPE‐PEG‐tagged Biotin was prepared in ethanol, achieving a concentration of 1 mm. A 100 µL aliquot of the exosome model sample was diluted five‐fold with Diluent C buffer and then incubated at 4 °C for 1 h upon mixing with 10 nmol of DSPE‐PEG‐Biotin in an additional 0.5 mL of Diluent C buffer. Simultaneously, NeutrAvidin (500 µg) was conjugated to the isothiocyanate‐grafted NPs by incubating at 37 °C for 1 h. 1% BSA was added to facilitate blocking. In the final step, the biotin‐labeled exosomes and freshly prepared NeutrAvidin‐decorated NPs were mixed together. At each time point, a 3 µL aliquot was extracted for nanoparticle tracking analysis, providing insights into the capturing efficacy.

### Synthesis of SiO_2_@BP@SiO_2_

The obtained SiO_2_ NPs underwent initial encapsulation with BP nanofilm on the surface facilitated by the adhesion of polydopamine. A reaction sample comprising 10 mL consisted of 2 mL of SiO_2_ nanoparticle dispersion (0.3 g mL^−1^), 6.5 mL of Tris·HCl buffer (pH 8.5), and 1.5 mL of dopamine hydrochloride (13.3 mg mL^−1^). The mixture was agitated for 6 h in an open glass vessel, resulting in a polydopamine coating on the NPs clusters and a noticeable change in solution color to black. After removing the supernatant, the NPs were ultrasonically dispersed in 10 mL of BP solution (1 mg mL^−1^) and stirred for 6 h, resulting in the formation of the SiO_2_@BP composite. After washing with water, the composite underwent silica deposition using the modified Stöber approach, ultimately yielding the designed SiO_2_@BP@SiO_2_ structure.

### Photothermal dPCR

The entire pool of small RNA within exosomes was extracted using the Tissue miRNA Kit (Vazyme, RC201) and eluted into 60 µL of water. After DNA degradation, a 50 µL reaction was prepared by adding 4 µL of reverse transcription master mix and 2 µm of stem‐loop primer, followed by incubation at 50 °C for 12 min and then at 85 °C for 5 min. Subsequently, 10 µL of cDNA was combined with a 40 µL PCR reaction comprising 0.3 µm of each primer, TaqMan probe, nanocomposite, 0.5 µL of BSA, and 0.5 µL of Tween 20. Employing a millipede‐structured microfluidic chip, the premixed solution was split into droplets of desired size in HFE‐7500 oil containing Krytox surfactant. Driven by the Arduino program, typical thermal cycles were executed: 95 °C for 1 min, followed by 32 cycles of 95 °C for 30 s, 60 °C for 60 s, and 72 °C for 60 s. Finally, the percentage of fluorescent microcarriers was quantified using a scanning confocal microscope. All experiments were conducted in triplicate.

### Statistical analysis

All quantitative characterizations were described as means with standard deviations (*n* = 3). *p*‐values were calculated by one‐way ANOVA with Tukey's multiple comparisons test. Statistical significance was defined as *p* ≤ 0.05, and the values were labeled with ^**^for *p* ≤ 0.01 and ^***^for *p* ≤ 0.001.

## Conflict of Interest

The authors declare no conflict of interest.

## Author Contributions

L.Z., R.P., and S.L. contributed equally to this work and are listed as co‐first authors. L.X.Z., D.X.W., G.Y., B.H. and F.F.Y. conceived the idea and designed the experiment; L.X.Z., R.P., S.Y.L., P.Y.C., G.L.W., D.M.H., K.L., and Y.C. conducted experiments and data analysis; L.X.Z. wrote the manuscript with the help from D.X.W., G.Y., B.H., and F.F.Y.

## Supporting information



Supporting Information

## Data Availability

The data that support the findings of this study are available from the corresponding author upon reasonable request.
