# Supplementary material for: Near‐Infrared‐Responsive Digital PCR‐Assisted Renal Cancer Exosomal miRNAs Insights and Regulation of Macrophage Polarization
Source: Adv Sci (Weinh). 2025 Aug 29;12(39):e09407. doi: 10.1002/advs.202509407 (PMC12533400; doi:10.1002/advs.202509407)
Supplement: Supplementary file 1 — Supporting Information [file ADVS-12-e09407-s001.docx]

**Supplementary Information for**

**Near-infrared-responsive Digital PCR-assisted Renal Cancer Exosomal miRNAs Insights and Regulation of Macrophage Polarization**

Lexiang Zhang^#^, Rokshana Parvin^#^, Siyue Lin^#^, Peiyu Chen, Gangling Wang, Dingmeng Hu, Ke Lin, Yun Cheng, Fangfu Ye*, Bing Han*, Gen Yang*, Dexuan Wang*

Corresponding author: Fangfu Ye, Bing Han, Gen Yang, Dexuan Wang

Email: fye@iphy.ac.cn; [kqbinghan@bjmu.edu.cn](mailto:kqbinghan@bjmu.edu.cn); gen.yang@pku.edu.cn; wangdexuan@wmu.edu.cn


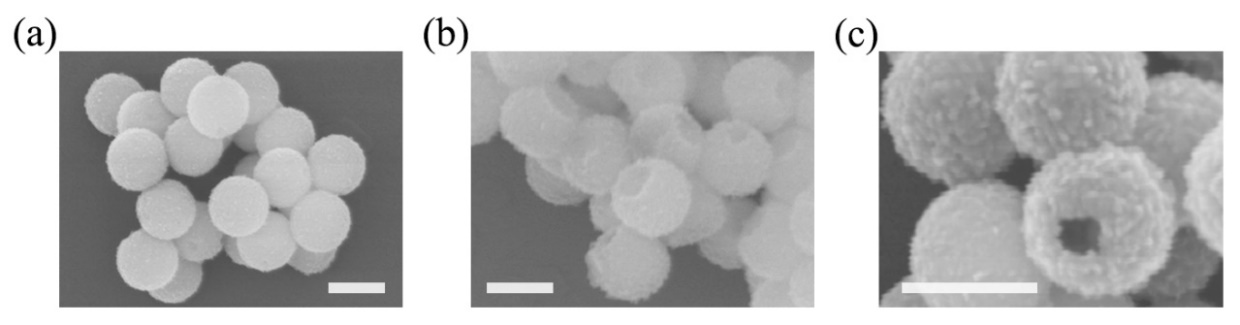


Figure S1. Different deposition states of the outer layer of magnesium silicate (a) A small degree of bumps. (b-c) Thick layer caused the NPs collapsed or became hollow. Scale bar, 250 nm.


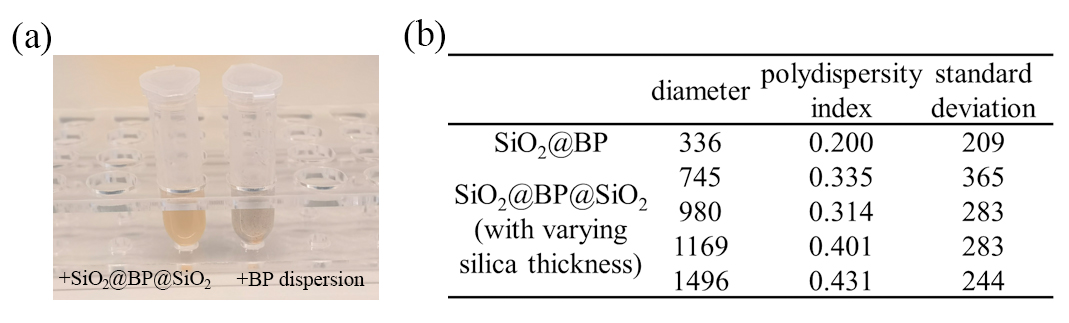


Figure S2. (a) Photograph of the dispersion of BP nanosheet and SiO_2_@BP@SiO_2_ in 7 % gelatin samples, respectively, after a few rounds of heating to 95 ℃. (b) Size distributions of NPs and composite with the polydispersity indexes and standard deviations





Figure S3. (a) Tiny patterns drawn on both sides of the chip that were used to align the upper and lower exposures. (b) Image showing the internal structure of the microfluidic chip.


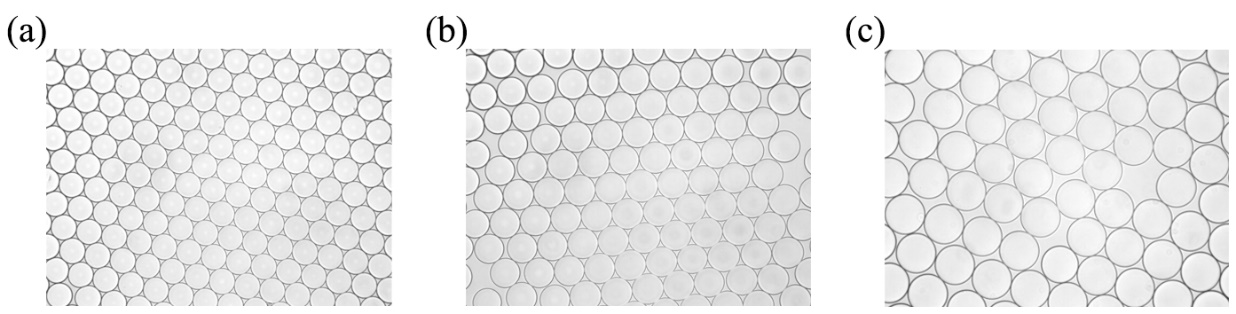


Figure S4. Monodispersed microcarriers produced with tunable diameters around 77-130 μm.


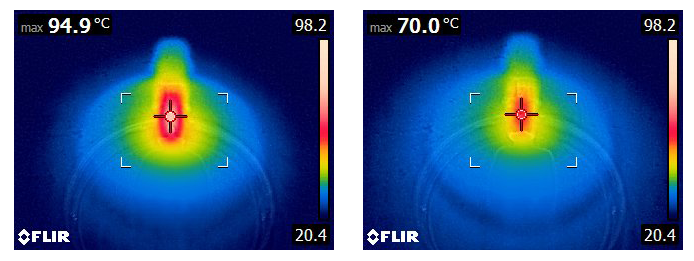


Figure S5. Thermal images of a model sample heated to the PCR temperature scope by NIR.


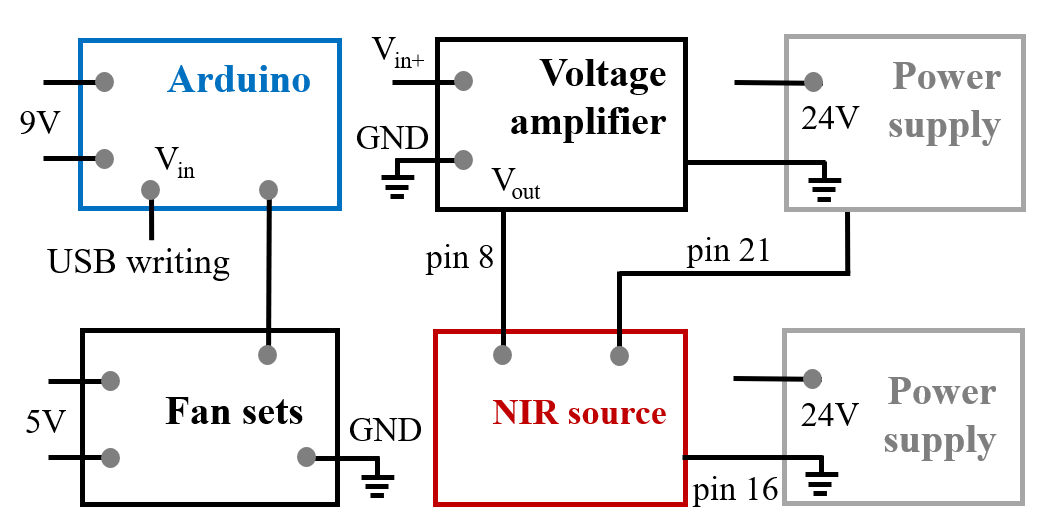


Figure S6. Wiring diagram of the programmable NIR control module.

Table S1. Sequencing of miRNA and mRNA targets and corresponding designed primers and probes.

| Name | Sequences (starting from 5’) |
| --- | --- |
| miR-30c | UGUAAACAUCCUACACUCUCAGC |
| miR-30c-RT1 | GTCGTATCCAGTGCAGGGTCCGAGGTATTCATCGGGTATACGGCGCACTGGATACGACGCTGAG |
| miR-30c-F1 | GTAAACATCCTACACTCTCAG |
| miR-30c-R1 | GTATCCAGTGCAGGGT |
| miR-30c-RT2 | GTCGTATCCAGTGCAGGGTCCGAGGTATTCTGGCTTAGAACGAGCGTTTGATAAGCACTGGATACGACGCTGAG |
| miR-30c-F2 | TGTAAACATCCTACACTCTC |
| miR-30c-R2 | CAGGGTCCGAGGTATT |
| miR-30c-RT3 | GTCGTATCCAGTGCAGGGTCCGAGGTATTCTAGCAGCGGTTAGTTAGCCTGCGTCTGATCCTGCGCACTGGATACGACGCTGAG |
| miR-30c-F3 | ACATCCTACACTCTCAGC |
| miR-30c-R3 | CAGGGTCCGAGGTATTC |
| miR-30c-RT4 | GTCGTATCCAGTGCAGGGTCCGAGGTATTCGCATCGACAGCTAATCGGGTATACGGCACCATTACTGCAGTAGATATTGGCACTGGATACGACGCTGAG |
| miR-30c-F4 | ACTCTCAGCGTCGTATC |
| miR-30c-R4 | CAGGGTCCGAGGTATT |
| miR-34 | UGGCAGUGUCUUAGCUGGUUGU |
| miR-34-RT | GTCGTATCCAGTGCAGGGTCCGAGGTATTCGATGGTCAAACGATTAGATTAACGATCGACAGTGGCGTACAGCTACATGGCACTGGATACGACACAACC |
| miR-34-F | CTTAGCTGGTTGTGTCGTATC |
| miR-34-R | GCAGGGTCCGAGGTATT |
| miR-210 | CUGUGCGUGUGACAGCGGCUGA |
| miR-210-RT | GTCGTATCCAGTGCAGGGTCCGAGGTATTCGAACTAAGAGTGCAAGATGGTCGCTCACTAGTTATGCTAGATGACAGCGGCACTGGATACGACTCAGCC |
| miR-210-F | AGCGGCTGAGTCGTATC |
| miR-210-R | GCAGGGTCCGAGGTATT |
| miR-126 | UCGUACCGUGAGUAAUAAUGCG |
| miR-126-RT | GTCGTATCCAGTGCAGGGTCCGAGGTATTCGCAACGGTTAGTTCACCTTCGTATGATGCTGATACAATGGCCAATAGAGCACTGGATACGACCGCATT |
| miR-126-F | GTGAGTAATAATGCGGTCGT |
| miR-126-R | GGTATTCGCAACGGTTAGT |
| STAT3-F | ATCACGCCTTCTACAGACTGC |
| STAT3-R | CATCCTGGAGATTCTCTACCACT |
| β-actin-F | GGGAAATCGTGCGTGACATTAAGG |
| β-actin-R | CAGGAAGGAAGGCTGGAAGAGTG |

Table S2. Significance analysis of different groups in Figures 5c, where “ns” represents not significant.


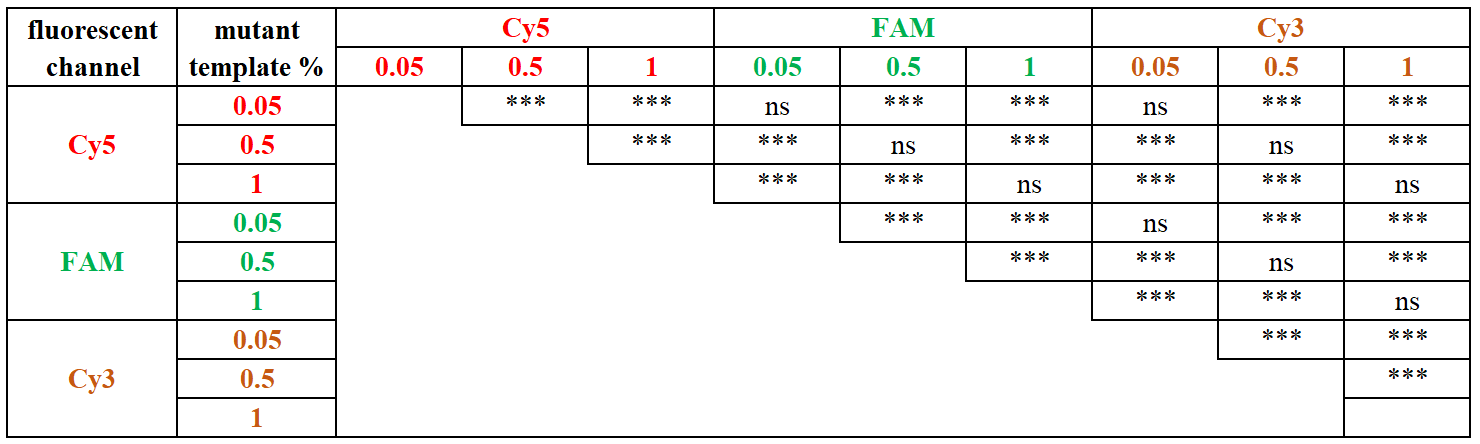


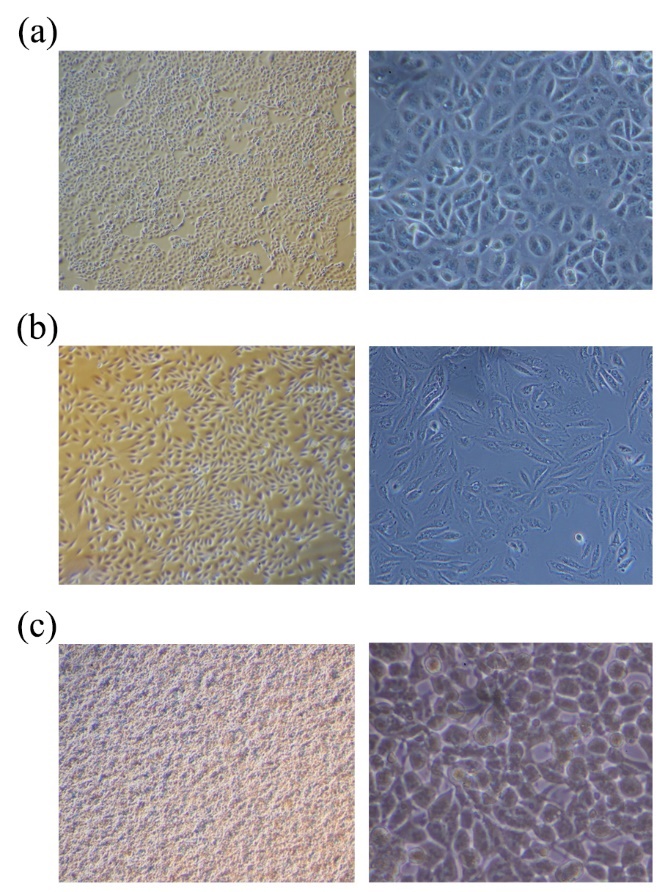


Figure S7. Cell states of (a) ACHN, (b) A498, and (c) HK-2.

Table S3. Significance analysis of different groups in Figures 5h.


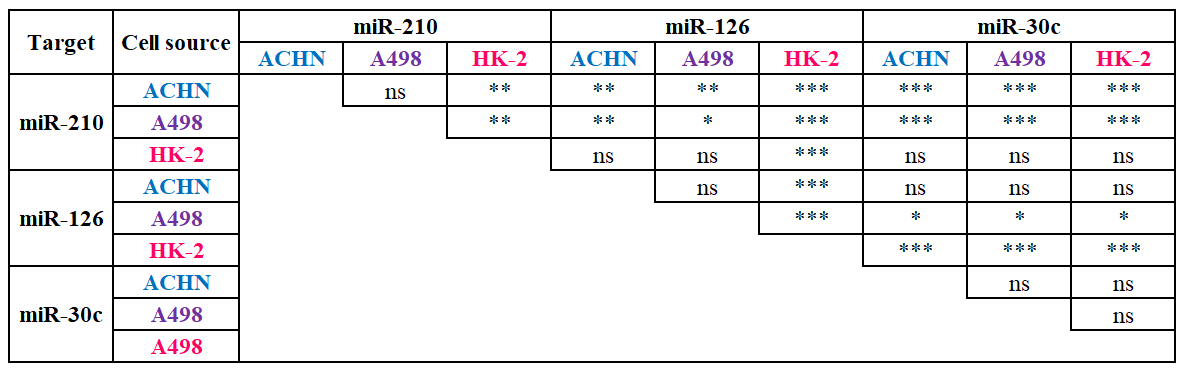


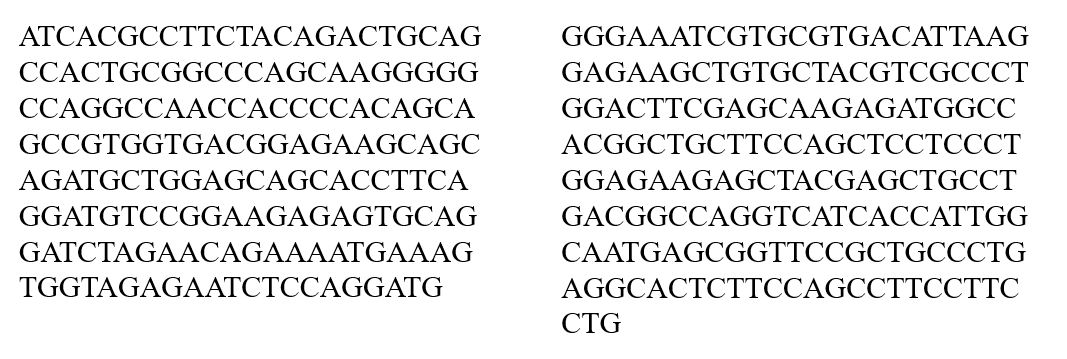


Figure S8. Sanger sequencing analysis of the products from STAT3 and β-actin genes, respectively.
